# Determinants of good or excellent work ability in a branch of the dutch military

**DOI:** 10.1007/s00420-025-02128-9

**Published:** 2025-02-25

**Authors:** Pablo M. Stegerhoek, Jesse van der Zande, Herman IJzerman, Evert A. L. M. Verhagen, Ehsan Motazedi, Caroline Bolling, P. Paul F. M. Kuijer

**Affiliations:** 1 Amsterdam Collaboration on Health and Safety in Sports, Department of Public and Occupational Health, Amsterdam Movement Sciences, Amsterdam UMC, Amsterdam, Netherlands; 2 Department of Public and Occupational Health, Amsterdam Public Health Research Institute, Amsterdam UMC, University of Amsterdam, Amsterdam, Netherlands; 3 Royal Netherlands Marechaussee, Health care section, Kalvermarkt 32, 2511 CB Den Haag, Netherlands

**Keywords:** Military, Police, Occupational health, Injury, Work ability

## Abstract

**Purpose:**

The Royal Netherlands Marechaussee, a branch of the Dutch Military, is characterised by a diverse range of mentally and physically demanding occupational tasks. The employability of the personnel depends on the balance between occupational demands and personal resources, which can be measured through the work ability score. Therefore, this study investigates personal and work-related determinants of work ability in a branch of the Dutch Military.

**Methods:**

We gathered cross-sectional data through a survey distributed among all operational Royal Netherlands Marechaussee personnel (n: 7,658). We used binomial logistic regression analysis to estimate the relationship between determinants in four domains (i.e., personal, workload, work characteristics, and work experience) and the dichotomised work ability scores (poor to moderate vs. good to excellent).

**Results:**

The survey had a 20% response rate with 1538 respondents. Our study included a slightly higher percentage of reservists and civilians than the Royal Netherlands Marechaussee’s workforce. Forty per cent of participants rated their work ability as poor or moderate. Good or excellent work ability was related to older age (> 50 years compared to < 29 years), lower physical workload, no shift work, less fatigue, more autonomy, task clarity, and social support.

**Conclusions:**

We found that 40% of survey respondents rated their work ability as low or moderate. In the future, factors like shift work, autonomy, task clarity, and social support may be used to improve work ability in this population.

**Supplementary Information:**

The online version contains supplementary material available at 10.1007/s00420-025-02128-9.

## Introduction

The Royal Netherlands Marechaussee (RNLM) is an independent branch of the Dutch armed forces concerned with border control, security and surveillance, and military policing (Defensie). The RNLM is the gendarmerie force of the Dutch military, and its personnel face various tasks, ranging from guarding structures and people of interest to managing border control at international airports (Defensie). These tasks are not just physically demanding due to long static load-carrying but also mentally demanding due to the required long-term focus and the continuous potential for extreme escalation. In addition, occupational demands vary from job to job and potentially from hour to hour. Keeping personnel employable for these diverse occupational demands is essential for the readiness of the RNLM.

Previous research by Stegerhoek et al. (2023) distinguished high and low occupational demand profiles in this population. A low perceived occupational demands profile was linked to high self-reported autonomy, experience, work support, and task clarity. The high perceived occupational demands profile was related to high physical and mental workload, physical and mental fatigue, and boredom (Stegerhoek et al. 2023). We used the same survey as Stegerhoek et al. (2023) for the current study. One of the main challenges regarding employability in this population is the balance between occupational demands and personal resources (Stegerhoek et al. 2023, 2024). To capture this balance, Ilmarinen (2007) proposed the work ability index, which they defined as the ability to handle occupational demands. Work ability is determined by personal well-being, health in the professional environment, and professional demands (Ilmarinen 2009). Work ability can be measured through the work ability index, which is a seven-dimension questionnaire (Ilmarinen 2009). However, the first item of the work ability index correlates highly with the total score and can, therefore also be used to measure work ability independently (Ahlstrom et al. 2010).

Previous work has investigated work ability in military and police contexts. For example, Barreto et al. (2019) found that 10% of the Brazilian military police force had poor work ability, 29% moderate, 35% good, and 26% had excellent work ability. Additionally, in their sample, being obese and working more than eight hours per day was related to poor work ability (Barreto et al. 2019). Goedhard and Goedhard (2005) found that 14% of a Dutch army unit had a poor to moderate score on the work ability index (Goedhard and Goedhard 2005). In this sample, work ability was negatively associated with age and perceived work stress. In the police context, Velasco-Garrido et al. (2022) found that work ability can be improved via a remodelled shift schedule. Semeijn et al. (2019) found no association between resilience and the work ability in Dutch Police officers. Interestingly, Soininen and Louhevaara (2000) found that an improvement in the work ability during a three-year police training was associated with work satisfaction but not physical fitness. To our knowledge, no study has investigated determinants of work ability in a gendarmerie corp.

Given its usefulness in capturing the balance between occupational demands and personal resources in other occupational settings, work ability may be a valuable tool in the military context. This study investigates work ability and its organisational and personal determinants in a gendarmerie corp.

## Method

### Design

This was a cross-sectional survey study. Given the nature of this study, the Dutch Military Medical Authority has approved our research (DOSCO 2021002986). We provided participants with information regarding confidentiality and anonymous data management, and each participant provided digital informed consent before gaining access to the questionnaire. We did not provide any financial or other incentives for participation. Stegerhoek et al. (2023) used data from the same survey in their study regarding occupational demand profiles.

### Participants and setting

We collected data at the RNLM in September of 2021 through a questionnaire we distributed via the RNLM intranet; the questionnaire was available for five weeks. To facilitate participation, we employed a three-step strategy: first, the highest-ranking commanding officer (Lieutenant-general) emailed all employees an invitation to complete the questionnaire. Next, we asked the brigade general to bring the questionnaire to the attention of their employees. Finally, after four weeks, we sent out a reminder via email. Participants had the opportunity to fill in the questionnaire during working hours.

In total, 7,658 RNLM employees received the questionnaire. These employees were employed in one of three branches: the staff, ‘*The National Centre for Training and Expertise’* (OTC), and *‘The National Tactical Command’* (LTC). The staff supports the commanding officer and consists of various departments. Even though the staff has the highest number of civilians employed in the three branches, most employees are military personnel. The OTC is the RNLM’s training centre, where recruits receive 12-week basic military training and 16-week theoretical education. Most employees at the OTC are military personnel. The LTC consists of 25 brigades located throughout the Kingdom of the Netherlands. Their responsibilities vary depending on the location of the brigade. The LTC is mainly comprised of military personnel.

### Measures

The items in the survey were designed to provide a complete understanding of the tasks and the accompanying workload for all RNLM personnel. To enable a broad understanding, we used items across five dimensions: (1) Personal, (2) Workload, (3) Work Ability, (4) Work Activities, and (5) Work Experience.

### Personal factors

The survey contained the following items in the personal domain: gender, having children (yes/no), age (years), years of experience in the military, years of experience in the RNLM, years in the current position, and rank (Marechaussee, Petty officer, Officer, Chief Officer, no rank). To ensure anonymous data collection, we registered participants’ age categories (i.e., ≤ 29 Years, 30–39 Years, 40–49 Years, > 50 Years).

### Workload

The survey’s workload domain consisted of two items: mental workload and physical workload. Both items were scored on a Borg-like scale of 0 to 10, with zero indicating minimal workload and 10 indicating maximum workload (Borg 1998).

### Work activities

We based the work characteristics part of the questionnaire on the Experience and Evaluation of Work Questionnaire (Dutch: VBBA) (Van Veldhoven and Broersen 2003). The items concerned time spent sitting, time spent driving, time spent walking, and time spent standing, all measured on a 5-point scale (Rarely -Sometimes – Frequently – Often – Almost Always). Furthermore, items concerned wearing a bulletproof vest (yes/no), wearing a weapon belt (yes/no), shift work (yes/no), working overtime (yes/no), hours on contract, and being a manager (yes/no).

### Work experience

We based the content of the work experience part of the questionnaire on the results of a qualitative study in the same population by Stegerhoek et al. (2024). It contained items regarding boredom, connectedness, mental fatigue, physical fatigue, autonomy in execution, autonomy in planning, peer support, support from a superior, task clarity, and clarity regarding responsibilities, which were all measured on a 5-point scale (Rarely -Sometimes – Frequently – Often – Almost Always). Next, we summed autonomy in execution and autonomy in planning (1–10), peer support and support from superiors, and task clarity and clarity regarding responsibilities (1–10) and analysed these as continuous variables.

### Work ability

Work ability was the dependent variable in this study, and it is a broadly used concept that describes how well employees can perform (Ilmarinen 2009). Previous work showed that poor work ability is related to reduced participation and early retirement (Burdorf et al. 2005; Van den Berg et al. 2008). Work ability can be measured using the work ability index, a questionnaire that measures seven aspects of work ability (Ilmarinen 2007, 2009). The WAI’s first item, the Work Ability Score (WAS), can also be used individually and is shown to be an excellent alternative to the complete WAI (El Fassi et al. 2013). The work ability score concerns the respondent’s current work ability to perform their work in relation to their all-time best; it ranges from 0 (completely unable to work) to 10 (workability at lifetime best) (Roelen et al. 2014). Research shows that the work ability score is accurate and reliable for measuring work ability (Ahlstrom et al. 2010; El Fassi et al. 2013; Gupta et al. 2014; Roelen et al. 2014). The designers of the method suggest the same categorisation as for the WAI, namely: poor (0–5 points), moderate (6, 7), good (8, 9), and excellent (10) (Gould et al. 2008).

### Statistical analysis

We used R, in R studio (version: 2022.7.0.548) (R development core team 2022) for all statistical analyses. First, we summarised all LTC, OTC, and staff demographic data and compared the personnel-type distribution in our sample to the distribution of the entire RNLM to verify its representativeness. Next, we calculated the percentage of participants in each predefined work ability category. The dataset contained missing values for the variables ‘travel time,’ ‘years of employment in the military,’ and ‘years of employment at the RNLM,’ resulting in an overall missing data percentage of 15%. We used a complete case analysis.

To answer our research question, we computed the work ability score as a dichotomous variable: poor (0–5) to moderate (6–7) versus good (8–9) to excellent (10) (González-Domínguez et al. 2024). We used logistic regression analysis to estimate univariable relationships between the previously mentioned potential determinants of work ability from each domain and the dichotomised work ability score. Furthermore, we used backwards and forward variable selection procedures based on improvements of the Akaike Information Criterion (AIC) to determine the most parsimonious model for each of the four domains (i.e., personal, workload, work activities, and work perception) (Chakrabarti and Ghosh 2011). The final multivariable models for each domain were built using the union of all predictors reported by the backward and forward selection procedures. Finally, we employed the least absolute shrinkage and selection operator (LASSO) regression to identify the most relevant determinants of work ability based on the lowest lambda value from the complete data set (all domains) and built a logistic regression model using these variables (Zhang et al. 2020). We reported odds ratios and 95% confidence intervals for each model and reported the Area Under the Curve (AUC) for the complete model.

Before we ran the variable selection procedure, we checked for multicollinearity using the variance inflation factor (VIF). If the VIF was higher than 5, we removed the variable from the model. Finally, we performed a sensitivity analysis on the final models to check for the robustness of the results to the type of personnel (military vs. civilian).

## Results

### Demographics and work ability

Of the 7,658 RNLM employees who received the questionnaire, 1,538 (20%) responded. The gender ratio was similar between our sample and the RNLM population, but our sample consisted of more civilians and fewer reservists. Table 1 shows the characteristics of the study population. Nine per cent of participants (*n* = 138) rated their work ability as poor (0 to 5), 31% (*n* = 478) as moderate (6 or 7), 52% (*n* = 787) as good (8 or 9), and 8% (*n* = 116) showed excellent (10) work ability.

**Table 1:** Descriptive data for all units

		LTC (*n = 1135)*		OTC (*n = 182)*		Staff (*n = 221)*		Total (*n = 1538)*	
		n	%	n	%	n	%	n	%
Work ability*	Poor and Moderate	474	42%	64	37%	78	36%	616	41%
	Good and excellent	652	58%	111	63%	140	64%	903	59%
Sex	Male	909	80%	133	73%	136	62%	1178	77%
	Female	217	19%	42	23%	82	37%	341	22%
	Transgender	2	0%	1	1%	0	0%	3	0%
	Prefer not to say	7	1%	6	3%	3	1%	16	1%
Age	≤ 29 Years	257	23%	33	18%	20	9%	307	20%
category	30–39 Years	335	30%	45	25%	46	21%	426	28%
	40–49 Years	261	23%	48	26%	55	25%	364	24%
	> 50 Years	282	25%	56	31%	100	45%	438	28%
Children	Yes	702	62%	119	65%	145	66%	966	63%
	No	433	38%	63	35%	76	34%	572	37%
Type of Employee	Military	1000	88%	156	86%	100	45%	1256	82%
	Civilian	125	11%	25	14%	117	53%	267	17%
	Reservist	10	1%	1	1%	4	2%	15	1%
Rank	Marechaussee	76	7%	22	12%	3	1%	101	7%
	Petty officer	711	63%	88	48%	15	7%	814	53%
	Officer	216	19%	44	24%	77	35%	337	22%
	Chief officer	8	1%	3	2%	9	4%	20	1%
	No Rank/ Not known	124	10%	25	14%	117	53%	266	17%
Overtime	Yes	766	67%	79	43%	135	62%	980	64%
	No	369	33%	103	57%	84	38%	556	36%
Managerial position	Yes	449	40%	64	35%	42	19%	555	36%
	No	686	60%	118	65%	177	81%	981	64%
Body Armor*	Yes	438	43%	15	10%	1	1%	454	36%
	No	572	57%	142	90%	102	99%	816	64%
Irregular Shifts	Yes	526	46%	9	5%	4	2%	539	35%
	No	609	54%	173	95%	215	98%	997	65%

LTC: National Tactical Command, OTC: National Centre for Training and Expertise

Note. These data were also reported in Stegerhoek PM, Van Der Zande J, Bolling C, IJzerman H, Verhagen E, Kuijer P. Royal Netherlands Marechaussee Personnel’s Self-Perceived Occupational Demand Profiles: A Latent profile analysis shows the “Good” versus the “Bad.” Military Medicine [Internet]. 2023 Mar 25;188(11–12):e3575–82. Available from: 10.1093/milmed/usad077; *A different N due to non-response

### Personal factors

Years of experience in the military had a VIF of > 7.00, so we removed this variable from the multivariable analysis. In the univariable analysis of the variables in the personal domain, more years of experience in the military (OR = 1.01; 95% CI 1.00–1.02) and was significantly associated with higher chances of good to excellent work ability scores. In contrast, the age group of 30–39 years had lower chances of good to excellent work ability scores compared to the reference group of < 29 years (OR (> 30–39 vs. < 29) = 0.65; 95% CI 0.46–0.91). Age was selected in the final multivariable model for the personal domain. In this model, age showed a significant positive association with a good to excellent work ability score, with the odds of good work ability being higher for someone 50 years or older compared to someone younger than 30 (OR = 1.60; 95% CI 1.18, 2.16) (Table 2).

**Table 2:** Uni- and multivariable logistic regression results for the effect of the personal and workload items on the work ability score(poor (0–5) to moderate (6–7) versus good (8–9) to excellent (10))

		Odds Ratio (95% CI) for good to excellent work ability; Univariable Analysis	Odds Ratio (95% CI) for good to excellent work ability; Multivariable Analysis
Personal Characteristics			
Sex (Female)		1.11 (0.84–1.46)	
Not having children (versus having children)		0.94 (0.74–1.18)	
Age Category	< 29	Ref	Ref
	30–39	0.65 (0.46–0.91) *	0.77 (0.57–1.04)
	40–49	1.11 (0.78–1.57)	1.23 (0.9–1.68)
	> 50	1.36 (0.97–1.90)	1.60 (1.18–2.16)*
Years in the military (per year increase)		1.01 (1.00–1.02) *	
Years in at the RNLM (per year increase)		1.01 (1.00–1.02)	
Years at current positions (per year increase)		1.02 (0.99–1.05)	
Rank	No Rank/ Not known	Ref	
	Marechaussee	1.00 (0.63–1.58)	
	Petty officer	1.17 (0.71–1.93)	
	Officer	1.40 (0.49–4.41)	
	Chief officer	1.15 (0.69–1.92)	
**Workload**			
Physical Workload (0–10, per point increase)		0.87 (0.83–0.91) ***	0.91 (0.87–0.95) ***
Mental Workload (0–10, per point increase)		0.83 (0.79–0.87) ***	0.85 (0.81–0.90) ***

RNLM: Royal Netherlands Marechaussee, CI: Confidence Interval,

* = *p* < 0.05, *** = *p* < 0.001

### Workload

Lower mental and physical workload were significantly associated with good to excellent work ability in the univariable analysis (Table 2). In the multivariable analysis, both physical and mental workload remained in the model based on the AIC. A one-point improvement in physical workload (OR = 0.91; 95% CI 0.87–0.95) and mental workload (OR = 0.85; 95% CI 0.81–0.90) decreased the odds of good to excellent work ability (Table 2).

### Work activity

In the work activity domain, not wearing a bulletproof vest, not wearing a weapon belt, and not doing shift work were significantly associated with good to excellent work ability in the univariable analyses (Table 3). However, the best model found through stepwise selection based on the AIC included shift work and time spent standing. Participants who did not work in shifts had 1.55 times greater odds for good work ability than participants who did work shifts (OR = 1.55; 95% CI 1.23–1.96). Participants who stood frequently (OR = 1.59; 95% CI 1.12–2.24) or often (OR = 1.76, 95% CI 1.16–2.65) had greater odds for good or excellent work ability than participants who rarely ever stood (Table 3).

**Table 3:** Uni- and multivariable logistic regression results for the effect of the work activity items on the work ability score (poor (0–5) to moderate (6–7) versus good (8–9) to excellent (10))

		Odds ratio (95% CI) for good to excellent work ability; Univariable Analysis	Odds ratio (95% CI) for good to excellent work ability; Multivariable Analysis
Work activity			
Time spent Standing	Almost Never	Ref	
	Sometimes	1.11 (0.78–1.58)	1.26 (0.9–1.76)
	Frequently	1.23 (0.86–1.75)	1.59 (1.12–2.24)*
	Often	1.29 (0.85–1.96)	1.76 (1.16–2.65)*
	Almost Always	1.02 (0.54–1.94)	1.36 (0.74–2.52)
Time spent Driving	Almost Never	Ref	
	Sometimes	0.94 (0.70–1.27)	
	Frequently	0.80 (0.58–1.11)	
	Often	0.96 (0.69–1.36)	
	Almost Always	0.91 (0.54–1.56)	
Time spent Walking	Almost Never	Ref	
	Sometimes	0.88 (0.59–1.31)	
	Frequently	0.90 (0.60–1.33)	
	Often	1.04 (0.66–1.65)	
	Almost Always	0.71 (0.37–1.36)	
Time spent Sitting	Almost Never / Sometimes	Ref	
	Frequently	1.34 (0.73–2.46)	
	Often	1.42 (0.80–2.53)	
	Almost Always	1.26 (0.70–2.26)	
Not wearing a bulletproof vest (versus wearing a bulletproof vest)		1.37 (1.08–1.75) *	
Not wearing a weapon belt (versus wearing a weapon belt)		1.33 (1.05–1.68) *	
Not doing shiftwork (versus doing shiftwork)		1.55 (1.23–1.96) ***	1.55 (1.23–1.96) ***
Not working overtime (versus working overtime)		1.09 (0.87–1.38)	
Working Hours (per hour increase)		1.00 (0.97–1.03)	
Not having a managerial position (versus having a managerial position)		1.03 (0.82–1.29)	

CI: Confidence Interval, * = *p* < 0.05, *** = *p* < 0.001

### Work experience

In the work experience domain, boredom, connectedness, physical fatigue, mental fatigue, autonomy, social support, and task clarity were significantly associated with good or excellent work ability scores (Table 4). The stepwise variable selection procedure based on improvement in AIC identified a model with only physical fatigue, mental fatigue, autonomy, work support, and task clarity. In this model, physical and mental fatigue showed strong negative associations with the work ability score. Almost always feeling physical fatigue resulted in smaller odds of good or excellent work ability (OR = 0.23; 95% CI 0.08–0.61), and almost always feeling mental fatigue resulted in smaller odds of good or good or excellent work ability (OR = 0.09; 95% CI 0.04–0.22). In contrast, autonomy (OR = 1.11, 95% CI 1.04–1.19), social support (OR = 1.21, 95% CI 1.13–1.29), and task clarity (OR = 1.14, 95% CI 1.07–1.23) were associated with good or excellent work ability (Table 4).

**Table 4:** Uni- and multivariable logistic regression results for the effect of the work experience items on the work ability score (poor (0–5) to moderate (6–7) versus good (8–9) to excellent (10))

		Odds ratio (95% CI) for good to excellent work ability; Univariable Analysis	Odds ratio (95% CI) for good to excellent work ability; Multivariable Analysis
Work experience			
Boredom	Almost Never	Reference	
	Sometimes	0.75 (0.58–0.96) *	
	Frequently	0.49 (0.30–0.79) **	
	Often	0.57 (0.33–1.01)	
	Almost Always	0.70 (0.26–1.88)	
Connectedness	Almost Never	Reference	
	Sometimes	2.27 (0.82–7.37)	
	Frequently	2.53 (0.94–8.01)	
	Often	3.95 (1.50–12.29) **	
	Almost Always	7.23 (2.74–22.59) ***	
Mental Fatigue	Almost Never	Reference	
	Sometimes	0.29 (0.14–0.55) ***	0.36 (0.17–0.70) **
	Frequently	0.17 (0.08–0.32) ***	0.31 (0.14–0.61) **
	Often	0.12 (0.05–0.22) ***	0.25 (0.12–0.51) ***
	Almost Always	0.03 (0.01–0.06) ***	0.09 (0.04–0.22) ***
Physical Fatigue	Almost Never	Reference	
	Sometimes	0.76 (0.56–1.03)	0.90 (0.65–1.25)
	Frequently	0.36 (0.25–0.51) ***	0.52 (0.35–0.77) **
	Often	0.25 (0.17–0.39) ***	0.49 (0.30–0.79) **
	Almost Always	0.07 (0.03–0.17) ***	0.23 (0.08–0.61) **
Autonomy (1–10, per point increase)		1.23 (1.16–1.30) ***	1.11 (1.04–1.19) **
Social Support (1–10, per point increase)		1.32 (1.24–1.40) ***	1.21 (1.13–1.29) ***
Task Clarity (1–10, per point increase)		1.34 (1.26–1.43) ***	1.14 (1.07–1.23) ***

CI: Confidence Interveal, * = *p* < 0.05,** = *P* < 0.01, *** = *p* < 0.001

### Complete model

The LASSO regression with 10-fold cross-validation identified 16 relevant variables based on a minimum lambda value of -4.92 to predict good or excellent work ability (Supplementary material 1). We used these 16 selected variables for the complete logistic regression model (Table 5). In this model, not doing shift work was associated with good or excellent work ability (OR = 1.44; 95% CI 1.03–2.01), while being between 30 and 39 years old was associated with poor or moderate work ability compared to being younger than 29 years old (0.61; 95% CI 0.39–0.94). Higher mental fatigue, physical fatigue, mental workload, physical workload, autonomy, and task clarity were associated with poor or moderate work ability (Table 5). The model’s AUC was 0.75, indicating acceptable discrimination.

**Table 5:** Complete multivariable logistic regression results (poor (0–5) to moderate (6–7) versus good (8–9) to excellent (10))

		Odds ratio (95% CI) for good to excellent work ability
Intercept		0.18 (0.02–1.56)
Not having children (versus having children)		1.23 (0.89–1.71)
Years in current position (per year increase)		1.02 (0.98–1.05)
Contract hours (per hour increase)		1.01 (0.98–1.04)
Not working overtime (versus working overtime)		0.76 (0.58–1.00)
Standing	Almost Never	Reference
	Sometimes	1.16 (0.76–1.77)
	Frequently	1.48 (0.91–2.39)
	Often	1.59 (0.90–2.83)
	Almost Always	1.59 (0.71–3.61)
Sitting	Almost Never / Sometimes	Reference
	Frequently	0.79 (0.38–1.63)
	Often	0.97 (0.48–1.95)
	Almost Always	1.23 (0.59–2.59)
Not doing shiftwork (versus to shiftwork)		1.44 (1.03–2.01)*
Age	< 29	Reference
	30–39	0.61 (0.40–0.94)*
	40–49	0.96 (0.59–1.57)
	> 50	0.91 (0.54–1.51)
Physical fatigue	Almost Never	Reference
	Sometimes	0.96 (0.68–1.36)
	Frequently	0.62 (0.41–0.96)*
	Often	0.61 (0.36–1.04)
	Almost Always	0.26 (0.09–0.72)*
Mental Fatigue	Almost Never	Reference
	Sometimes	0.42 (0.19–0.85)*
	Frequently	0.39 (0.17–0.83)*
	Often	0.32 (0.14–0.68)**
	Almost Always	0.12 (0.04–0.32)***
Connectedness	Almost Never	Reference
	Sometimes	1.30 (0.39–4.82)
	Frequently	1.33 (0.41–4.86)
	Often	1.35 (0.41–4.96)
	Almost Always	1.71 (0.49–6.63)
Physical workload (0–10, per point increase)		0.93 (0.87–0.98)**
Mental workload (0–10, per point increase)		0.93 (0.87–0.99)*
Autonomy (0–10, per point increase)		1.08 (1–1.16)*
Social support (0–10, per point increase)		1.1 (0.99–1.21)
Task clarity (0–10, per point increase)		1.23 (1.14–1.32)***
AUC		75.22%

CI: Confidence Interval, AUC: Area Under the Curve, * = *p* < 0.05, ** = *P* < 0.01, *** = *p* < 0.001

### Sensitivity analysis

The effect estimates regarding personal factors, workload, work activity, and work experience did not differ significantly between military and civilian personnel in the subdomain models. However, in the complete model, a higher physical workload was significantly associated with lower odds of having good or excellent work ability for civilian personnel (OR = 0.80; 95% CI 0.67–0.95), but not for military personnel (OR = 0.94; 95% CI 0.88–1.00).

## Discussion

This study found that 40% of RNLM survey respondents rated their work ability as poor or moderate. Additionally, we identified various personal, workload, work activity, and work experience-related factors that were associated with work ability.

### Age as a protective factor

In the current study, older age was related to a good or excellent work ability score, with the association becoming more substantial with the older age groups (Table 2). Interestingly, in the complete model, only participants in the age group of 30–39 years old showed lower odds of good or excellent work ability (Table 5). A previous systematic review by Van den Berg et al. (2008) showed that older age is typically associated with lower work ability scores, and a recent Umbrella review showed that age is linked to various adverse health outcomes (Stegerhoek et al. 2024). This negative relationship between age and work ability has been found in studies with hospital workers (Weigl et al. 2013), bus drivers (Kloimüller et al. 2000), and home care workers (Pohjonen 2001). A partial explanation for these contradictory findings may be found in the work by Ivie and Garland (2011), who found that previous military experience may benefit police officers with regard to handling occupational stressors. This suggests that the increased experience in the military, because of older age, may offer a protective effect against poor work ability. Alternatively, the increase in age may coincide with changes in rank and job description, resulting in work with more autonomy, lower physical workload, and less shiftwork, which are all related to higher work ability scores in the current study. This would also explain why, in the complete model, the older age categories were no longer associated with good or excellent work ability. Future research could explore the effect of age, autonomy, physical workload, and shift work on work ability in the military context.

### Autonomy, work support, and task clarity

Enhancing the ability of personnel to handle the mental occupational demands could be focused on increasing autonomy, social support, and task clarity, which are related to good to excellent work ability in our study. For example, Delahaij et al. (2014) showed that support for autonomy among military employees reduces the intention to quit. However, work support may be more ambiguous in the military context, given most militaries’ strictly hierarchical organisation characteristics. Woo et al. (2021) found that superior support protects Korean military officers against depression and Piotrowski et al. (2021) found that superior support positively influences police officers’ work engagement. Finally, Lang et al. (2007) showed that personnel in highly demanding circumstances benefitted from task clarity. They also highlight that task clarity is potentially easily modifiable through policies and education for managers (Lang et al. 2007). Therefore, these three domains, autonomy, work support, and task clarity, can potentially be modified to improve work ability in the military context. Future studies should investigate potential ways to modify these factors in this population.

### Shift work

As expected, since previous work extensively documented the adverse effects of shift work, we found a strong association between shift work and poor to moderate work ability (Rivera et al. 2020). Specifically, in the military police population, França et al. (2011) found that shift work is a critical source of stress. Nevertheless, shift work is often an inevitable part of the job, so efforts to aid personnel in handling the consequences of shift work well are paramount. Additionally, although shift work is often unavoidable in this context, there may be more favourable ways to arrange it (Stegerhoek et al. 2024). For example, switching to a fast rotation schedule, where the maximum number of consecutive shifts of the same sort is four, resulted in better health and work-life balance in shift workers (Bambra et al. 2008). Moreover, power naps during shift work can mitigate acute fatigue in emergency personnel (Martin-Gill et al. 2018). Future research could investigate if these interventions are feasible in the military context.

### Strengths and limitations

The strengths of this research include that, to our knowledge, this is the first study investigating determinants of work ability in a gendarmery corps. Furthermore, we used an instrument with validated items regarding workload, work characteristics, and work experience. Nevertheless, this study has several limitations. First, the study is cross-sectional, limiting the degree to which directional claims can be made about the findings. Furthermore, the current sample results from only a 20% survey response rate and contains more civilians and reservists than the RNLM population. In the context of the RNLM, a higher percentage of non-military personnel may result in a sample with less physically demanding occupational tasks. However, we performed sensitivity analyses to account for our sample’s higher proportion of civilians. Considering the high prevalence of poor or moderate work ability in the current sample, we cannot rule out the possibility of sampling bias. The magnitude of the relation between dependent and independent variables in the current sample may also be inflated due to common source bias. Finally, given that the RNLM’s tasks may differ from those of other military branches in different countries, researchers and practitioners should be careful in extending these results to other populations.

## Conclusion

The current study found that four out of ten RNLM survey respondents rate their work ability as poor to moderate. Good or excellent work ability is related to older age, less physical and mental workload, no shift work, less fatigue, and more autonomy, task clarity, and social support. The military’s hierarchical nature allows for potentially easy manipulation of social support, autonomy among personnel, and task clarity. Furthermore, with shift work being inevitable in this population, efforts should be made to minimise possible adverse effects. Future research should study work ability longitudinally in a military population.

## Electronic supplementary material

Below is the link to the electronic supplementary material.

Supplementary Material 1

## Data Availability

The data are available upon reasonable request.

## Declarations

### Disclaimer

The views expressed in the article are those of the research group and do not reflect the official positions or views of the Royal Netherlands Marechaussee.
