# Supplementary figures and images for: Determinants of good or excellent work ability in a branch of the dutch military
Source: Int Arch Occup Environ Health. 2025 Feb 25;98(2):233–42. doi: 10.1007/s00420-025-02128-9 (PMC11937055; doi:10.1007/s00420-025-02128-9)

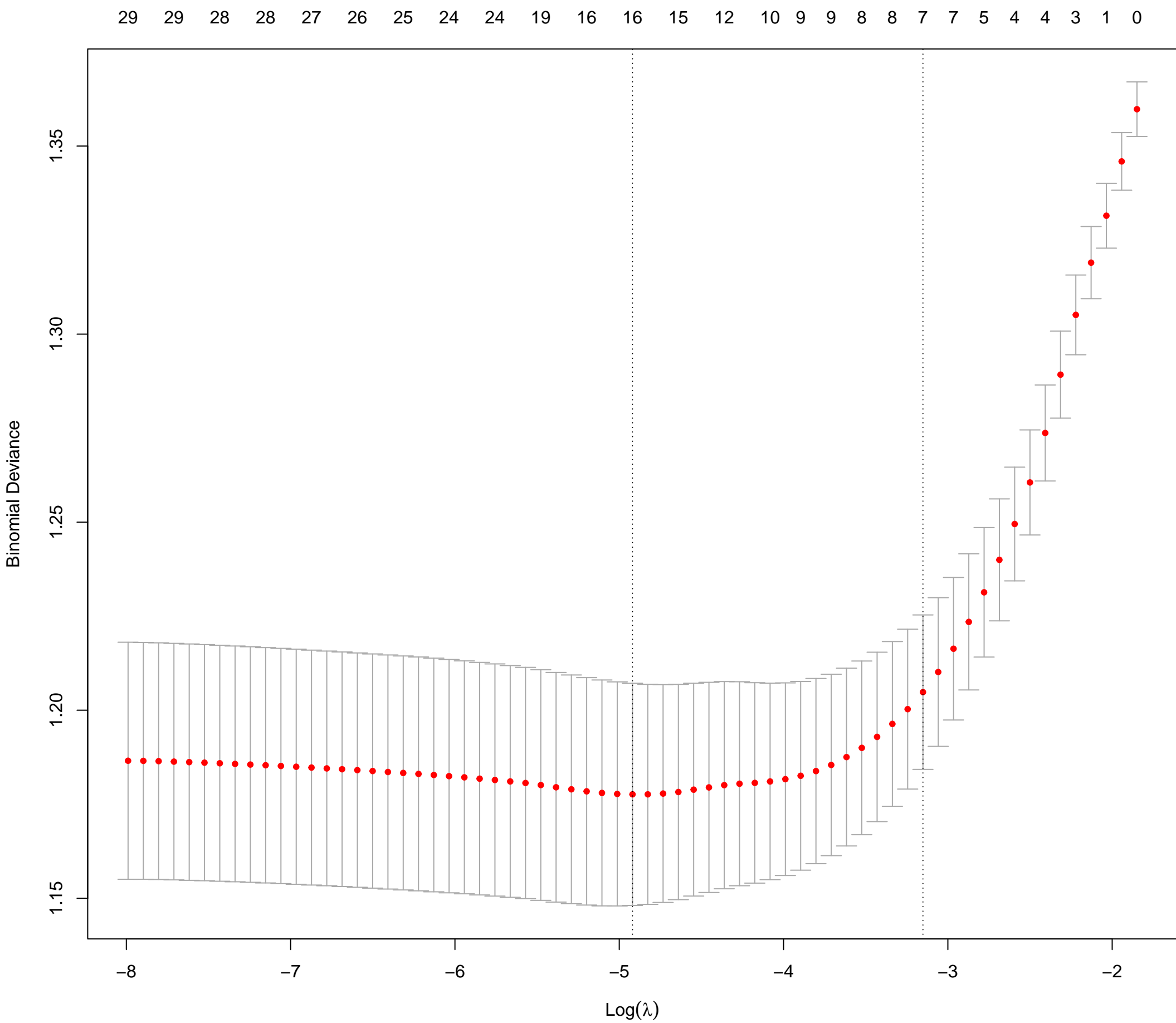

Supplement: Supplementary file 1 — Supplementary Material 1 [file 420_2025_2128_MOESM1_ESM.pdf]
